# Sphingolipids and plasma membrane hydrolases in human primary bronchial cells during differentiation and their altered patterns in cystic fibrosis

**DOI:** 10.1007/s10719-020-09935-x

**Published:** 2020-07-14

**Authors:** Nicoletta Loberto, Giulia Mancini, Rosaria Bassi, Emma Veronica Carsana, Anna Tamanini, Nicoletta Pedemonte, Maria Cristina Dechecchi, Sandro Sonnino, Massimo Aureli

**Affiliations:** 1 Dip. Biotecnologie Mediche e Medicina Traslazionale, Università degli Studi di Milano, LITA, Via Fratelli Cervi 93, Segrate, Milano 20090 Italy; 2 Section of Molecular Pathology, Department of Pathology and Diagnostics, University Hospital of Verona, 37126 Verona, Italy; 3 U.O.C. Genetica Medica, IRCCS Giannina Gaslini, 16147 Genova, Italy; 4 Section of Clinical Biochemistry, Department of Neurosciences, Biomedicine and Movement, University of Verona, 37134 Verona, Italy

**Keywords:** Primary cells, Bronchial cells, Sphingolipids, Hydrolases, Membrane domains, Cystic fibrosis, CFTR

## Abstract

Human primary bronchial epithelial cells differentiated *in vitro* represent a valuable tool to study lung diseases such as cystic fibrosis (CF), an inherited disorder caused by mutations in the gene coding for the Cystic Fibrosis Transmembrane Conductance Regulator. In CF, sphingolipids, a ubiquitous class of bioactive lipids mainly associated with the outer layer of the plasma membrane, seem to play a crucial role in the establishment of the severe lung complications. Nevertheless, no information on the involvement of sphingolipids and their metabolism in the differentiation of primary bronchial epithelial cells are available so far. Here we show that ceramide and globotriaosylceramide increased during cell differentiation, whereas glucosylceramide and gangliosides content decreased. In addition, we found that apical plasma membrane of differentiated bronchial cells is characterized by a higher content of sphingolipids in comparison to the other cell membranes and that activity of sphingolipids catabolic enzymes associated with this membrane results altered with respect to the total cell activities. In particular, the apical membrane of CF cells was characterized by high levels of ceramide and glucosylceramide, known to have proinflammatory activity. On this basis, our data further support the role of sphingolipids in the onset of CF lung pathology.

**Electronic supplementary material:**

The online version of this article (10.1007/s10719-020-09935-x) contains supplementary material, which is available to authorized users.

## Introduction

Sphingolipids (SLs) are bioactive lipids asymmetrically distributed at the external leaflet of the plasma membrane (PM) of all eukaryotic cells. They are characterized by a hydrophilic head group protruding toward the extracellular environment and by ceramide (Cer) a peculiar lipid moiety, deeply inserted into the membrane bilayer [1]. Cer is composed by a long chain base, the sphingosine, linked to a fatty acid of different length and saturation. Due to their chemical and physical properties SLs segregate in restricted membrane areas known as “lipid rafts”. These lipid domains, formed with the participation of cholesterol and a selected pattern of proteins, are macromolecular complexes involved not only in structural function, but also in the control of signalling transduction across the membrane [2].

In epithelial cells, PM organization is emphasized by cell polarization and thus it is possible to distinguish a basal plasma membrane domain facing the underlying tissue, a lateral domain contacting adjacent cells and an apical domain facing the exterior lumen.

The apical membrane of epithelial cells is typically enriched in SLs and cholesterol, so as to be considered as a lipid raft as a whole [3]. However, within apical membrane, the lateral distribution of lipids allows the organization in small domains which host specific membrane proteins and promotes the membrane curvature which enables to reach the specific apical structure of epithelial cells [4, 5].

During the different stages of cell cycle, SL pattern undergoes a continuous modulation in order to better adapt the membrane properties to specific requests. This aspect has been particularly studied in fundamental cell processes such as proliferation and differentiation [6].

By a systematic review of the literature clearly emerges that SLs play an important role also in the homeostasis of pulmonary cells. The lung is characterized by the presence of a complex pattern of SLs including Cer, phospho-SLs, neutral glycosphingolipids and gangliosides [7]. In particular, the preservation of SL rheostat composed by Cer, known to induce apoptosis, and sphingosine-1-phosphate, which promotes cell survival, is fundamental for the formation of lung structure at all stages of lung development, as well as the preservation of pulmonary physiology [8]. Furthermore, de novo synthesis of Cer has been linked to apoptotic endothelial cell death and impaired pulmonary barrier function [9, 10].

In the past ten years the role of SLs in the pathophysiology of cystic fibrosis (CF) has been also investigated. CF is one of the most common inherited disorder caused by mutations in a gene coding for the Cystic Fibrosis Transmembrane conductance Regulator (CFTR) protein [11]. The most frequent mutation is the deletion of phenylalanine 508 (F508del), which is present in approx. 80% of the alleles worldwide, with marked differences in its frequency depending on countries and ethnic groups [12].

A specific role of SLs seems to be played in the control of the inflammatory response to Pseudomonas aeruginosa (*P. aeruginosa*) infection. Indeed, bacterial infection of different CF bronchial epithelial cell lines leads to the activation of the PM associated SL-hydrolases, in particular the non-lysosmal β-glucosylceramidase (NLGase). These enzymes are responsible for the aberrant catabolism of SLs resulting in the production of Cer at the PM level [13–15].

Although several lines of evidence support the involvement of SLs in CF lung disease [16–18], their mechanistic role is still not completely understood. One of the reasons resides on the difficulty to collect data related to the SLs profile *in vivo*. However, also the more reliable *in vitro* model of airway epithelium represented by human primary bronchial cells differentiated at the air liquid interface, has never been studied for its SLs profile.

Here we reported the changes in SLs occurring during differentiation of human primary bronchial cells derived from non-CF subjects and from CF patients homozygous for F508del mutation. In addition, we depicted the differences between apical and basolateral membrane in terms of SL composition and distribution of SL hydrolases involved in the production of proinflammatory Cer.

## Results and discussion

### Sphingolipid pattern of human bronchial cells during different stages of in vitro airway epithelial differentiation is altered in cystic fibrosis

During differentiation, primary bronchial epithelial cells cultured at air liquid interface (ALI) can give rise to different cell types, such as ciliated, goblet, club and basal cells, that are peculiar of the airway epithelium. Different culturing conditions and use of specific stimuli can modify the expression of distinct cell types, as in the case of treatment with IL-4, a condition that mimics allergic and asthmatic inflammation, causing the differentiation of a subpopulation of bronchial epithelial cells towards goblet cells [19].

The differentiation process, analogously to that occurring in vivo, consists in three different phases:

1. *proliferative stage* - cells are grown in canonical supports (flasks) for the cell culture previously coated with collagen from rat tail in order to mimic the lung parenchyma. The cells at this stage recapitulate the staminal-like, basal cells of the airway epithelium, involved for instance in the airway epithelium turnover or in the repair of bronchial injury.
2. *early differentiation stage* - human primary bronchial cells are seeded on permeable supports (transwells) and cultured with a differentiating medium added both in the bottom and upper side of the chamber for 7–8 days. In this stage, bronchial cells start to develop the junction network needed to form an epithelium similarly to what occurs *in vivo*. In addition, the cell polarization occurs with the definition of the apical and basolateral portion.
3. *fully differentiation stage* - at the end of the early differentiation stage, the bronchial epithelium has developed a transepithelial resistance and the medium is removed from the upper side of the transwell. The differentiation of the epithelium continues under ALI condition for additional 7–8 days, when the bronchial epithelium is considered fully differentiated.

These three steps are fundamental for the formation of a functional respiratory epithelium and, as described above, they recapitulate the process that also occurs in vivo.

Since SLs play an important role in cell differentiation they were analysed after metabolic labelling with [1-^3^H]sphingosine. This radioactive SL precursor is rapidly taken up by the cells and inserted into the sphingolipid metabolic biosynthetic pathway. In addition, the catabolism of [1-^3^H]sphingosine produces tritium labelled ethanolamine, which is recycled for the biosynthesis of phosphatidylethanolamine (PE). We first analysed the total radioactive lipid pattern of three human bronchial epithelial cell cultures derived from non-CF subjects (HBE-WT) at all the three stages of *in vitro* differentiation.

**Fig. 1:**
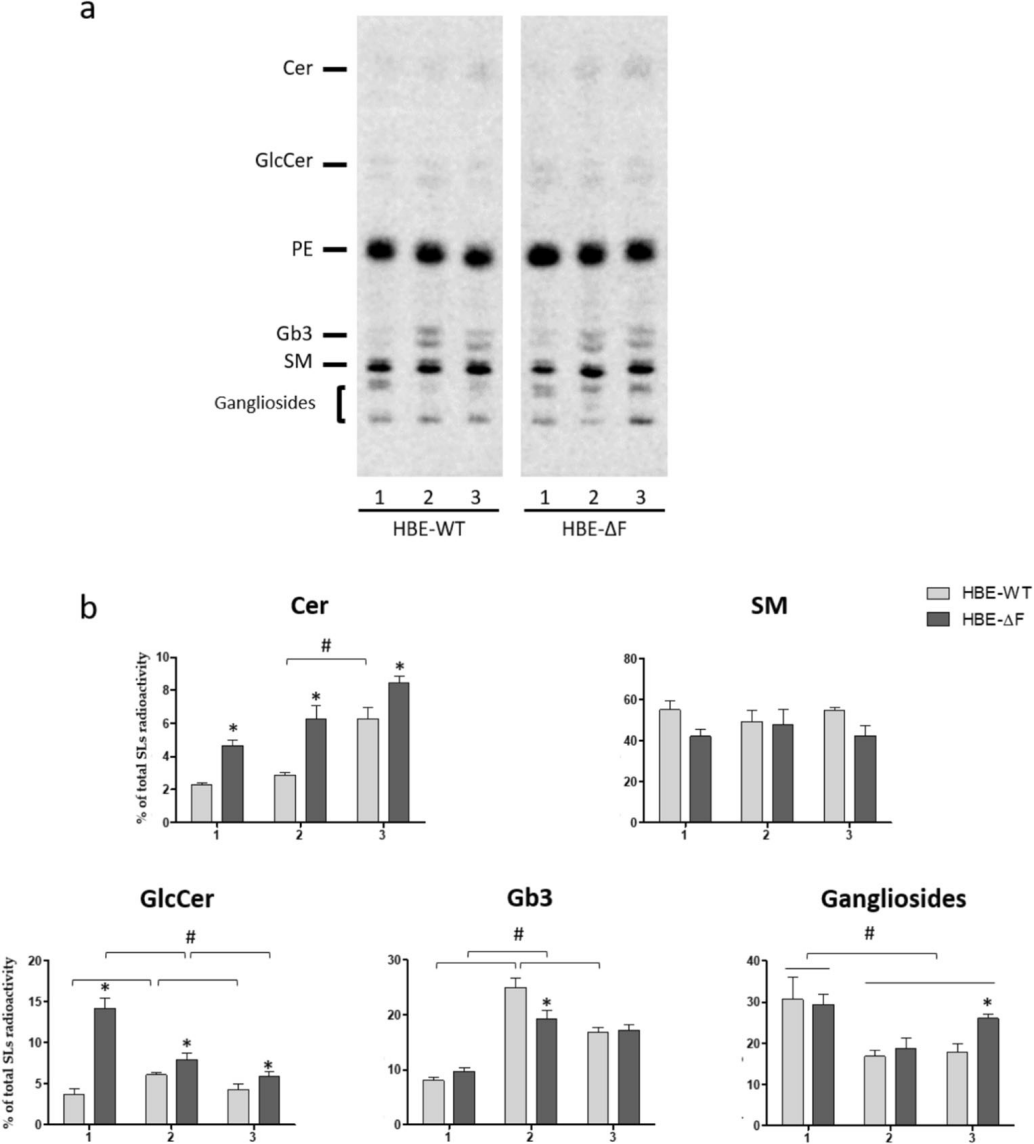
Sphingolipid pattern of human primary bronchial cells derived from non-CF subjects and CF patients at different stages of epithelial differentiation. Cell sphingolipids were metabolically labeled at the steady state using [1-^3^H]sphingosine as indicated in the "Methods" Section. **a** Representative digital-autoradiography of radioactive lipids of human primary bronchial cells derived from non-CF subjects (HBE-WT) and CF patients homozygous for F508del mutation (HBE-ΔF) at the stage of: proliferative cells (1), early differentiated cells (2), and fully differentiated cells at air liquid interphase (3). **b** Semi quantitative graph of sphingolipid species of three different non-CF and CF cultures. For each sphingolipid data are reported as percentage of total SLs incorporated radioactivity. Cer: ceramide, GlcCer: glucosylceramide, PE: phosphatidylethanolamine; Gb3: globotriaosylceramide, SM: sphingomyelin *p < 0.05 vs. HBE-WT, ^#^p < 0.02 vs. the indicated group

As shown in Fig. 1, over 50% of the total cell incorporated radioactivity is associated with PE and sphingomyelin (SM). SM is the main SL and its content does not change during the different stages of differentiation.

On the other hand, during differentiation we found an increased content of Cer and globotriaosylceramide (Gb3) which is paralleled by a decrease in glucosylceramide and gangliosides level. Of note, Gb3, a functional receptor for Shiga toxins, becomes the major SL in differentiated cells [20].

We performed the same analyses on cultures of human primary bronchial cells derived from three different CF patients homozygous for F508del mutation (HBE-ΔF). Interestingly, at all the stages analysed, CF cells are characterized by a higher content of Cer compared to HBE-WT. Several *in vitro* and *in vivo* studies demonstrated that Cer has a key role in the onset of inflammation in CF lungs [21], even if the time course of Cer accumulation in CF bronchi is still under debate. The new observation here reported suggests that increased Cer content in CF could be considered a feature of the disease since it is already present at the early stages of differentiation. Indeed, the highest levels of Cer were found in proliferative and early differentiated HBE-ΔF cells. In addition, CF cells also show an increased content of glucosylceramide, another important proinflammatory lipid (Fig. 1) [21, 22].

An alternative protocol for the commitment of primary human bronchial cells to bronchial epithelium is represented by the one developed by Coraux and collaborators (here after referred to as “Coraux method”) that allows to obtain an epithelium enriched in goblet cells that are responsible for the mucus production [23].

**Fig. 2:**
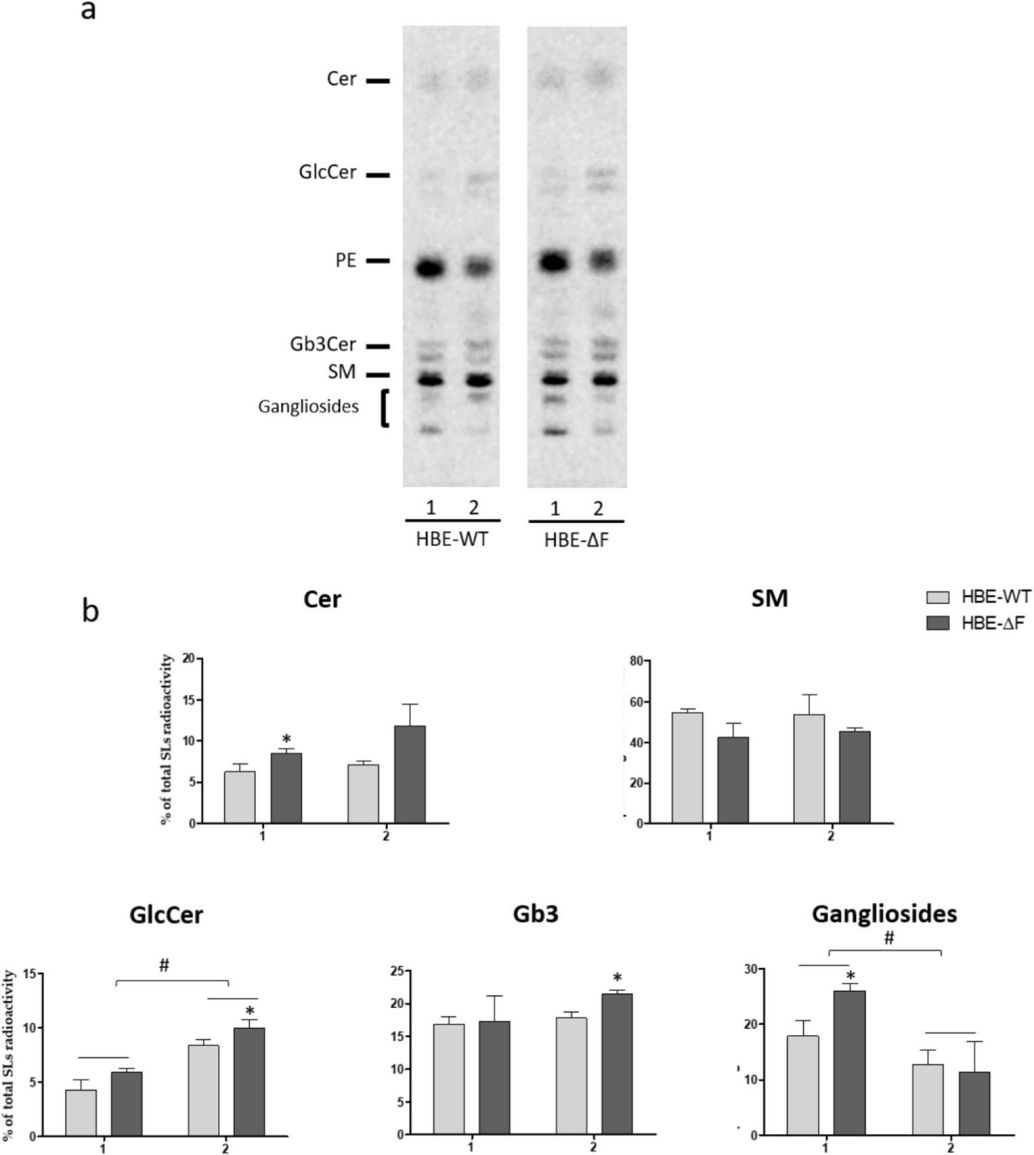
Sphingolipid pattern of human primary bronchial cells derived from healthy subjects and CF patients differentiated at air-liquid interface (ALI): epithelial vs. Coraux method. Cell sphingolipids were metabolically labeled at the steady state using [1-^3^H]sphingosine as indicated in "Methods" Section. **a** Representative digital-autoradiography of radioactive lipids of human primary bronchial cells derived from healthy subjects (HBE-WT) and CF patients homozygous for F508del mutation (HBE-ΔF) subjected to epithelial differentiation at ALI (1), or to Coraux differentiation at ALI (2). **b** Semi quantitative graph of sphingolipid species of three different non-CF and CF cultures differentiated by epithelial vs. Coraux method. For each sphingolipid data are reported as percentage of total SLs incorporated radioactivity. Cer: ceramide, SM: sphingomyelin, GlcCer: glucosylceramide, Gb3: globotriaosylceramide *p < 0.05 vs. HBE-WT, ^#^p < 0.01 vs. the indicated counterpart

We compared the SLs composition of HBE-WT and HBE-ΔF cells differentiated under ALI using the standard protocol or the Coraux method. As shown in Fig. 2, the SLs pattern of cells subjected to the two differentiation protocols differs only for an increased content of glucosylceramide followed by a decrease in complex gangliosides in the Coraux method with respect to the classical one. The reduced content of PE in cells differentiated with Coraux method suggests an increased recycling of tritiated sphingosine with respect to its catabolism under these experimental conditions (Fig. 2a). In CF cells differentiated by the Coraux method, we observed that Gb3 is significantly higher than in non-CF cells grown with the same protocol. Gb3, together with Gb4, has been reported to exhibit a TLR4-mediated proinflammatory activity and cause nephropathy in diabetic mice [24]. When cells are grown by the Coraux method, which induces the differentiation of a subpopulation of bronchial epithelial cells towards goblet cells, the originated epithelium results in excessive production and retention of mucus, particularly in CF deriving cells. This feature is common to several chronic airways diseases and related to an inflammatory state. For this reason, the difference observed in the Gb3 level between CF and non-CF cells let suggest a possible involvement of Gb3, in addition to Cer and glucosylceramide, in CF pathology.

Taken together these data, although obtained from cultures from only three non-CF and CF subjects, indicate that during the in vitro differentiation human bronchial epithelial cells undergo significant changes in the SLs pattern which are consistent with the epithelium formation, especially for the increased content of Gb3 in differentiated cells with respect to the proliferative stage. In addition, the increased content of Cer and glucosylceramide found in HBE-ΔF cells represent an additional evidence related to the possible involvement of these two lipids in the etiopathology of CF lung disease.

### The apical membrane of HBE-ΔF cells is characterized by an enrichment of proinflammatory sphingolipids and hydrolases involved in their production

As occurs in human bronchi, human bronchial epithelial cells fully differentiated in vitro at ALI undergo a specific three-dimensional polarization defining an apical membrane, which is that oriented in the lumen of the organ (upper side of the differentiation chamber), and a basolateral membrane which is that anchoring cell to the extracellular matrix (bottom of the chamber). Several hypotheses were formulated on the different chemical and physical properties of these two specialized membranes, since they must accomplish different functions. In particular, the apical membrane has to resist to dangerous insults, such as the oxidative stress and the dehydration. SLs, by the formation of the so-called lipids rafts, can organize specific portions of the PM with peculiar features in terms of membrane architecture, structure and control of the cell signalling [25]. Nevertheless, no data related to SLs distribution between the two membranes in polarized human bronchial cells are available so far. The main drawback for these studies is the viability of a methodology that allows to isolate portions of the apical or basolateral membrane with high grade of purification and with enough lipidic material to perform the analyses.

We addressed this issue exploiting the strategy that allows to obtain an enrichment of apical membrane portions based on biotin-streptavidin precipitation of all the proteins residing in the outer leaflet of the apical membrane in lipid-domain preserving conditions [26]. HBE-WT and HBE-ΔF cells fully differentiated at ALI were fed from the apical and basolateral side with [1-^3^H]sphingosine to metabolically label at the steady state cell SLs. This approach allows to increase the sensitivity of SL detection. After that, the proteins of the outer leaflet of the apical membranes were biotinylated. The protocol of biotinylation at 4 °C avoids biotin internalization and the resistance of the epithelium blocks the biotin derivative in the upper chamber. These conditions allow the selective labelling of the proteins of the outer leaflet of the apical membrane.

Subsequently cells were lysed as described in the "Methods" Section in order to preserve the lipidic environment of the proteins. Post nuclear supernatant (PNS) was then precipitated using streptavidin magnetic beads. The lipids from PNS, streptadivin-precipitate (SP), and supernatant obtained after precipitation (SN) were extracted and radioactive lipids analysed as described in the "Methods" Section. As shown in supplementary Fig. 1, panel A, the streptavidin-precipitates from HBE-WT and HBE-ΔF were obtained with similar efficiency as demonstrated by the HRP-streptavidin staining (upper image). The biotinylated proteins were almost recovered in the SP fraction, whereas a very low amount of biotinylated proteins was present in SN.

The western blot performed using the specific antibody for β-actin, performed on the same PVDF, allowed to detect only a faint signal in the SP fraction, thus proving that apical membrane biotinylated proteins were efficiently pooled down by streptavidn precipitation. The presence of β-actin in SP samples is not an unexpected result. As known, many PM proteins interact with cytoskeleton and this is particularly important in polarized cells to maintain the proper features of the apical and baso-lateral membranes [27]. For this reason, the presence of a small amount of β-actin in the SP fraction could be due to a detergent resistant interaction between apical PM proteins and the cytoskeleton.

The SL profile associated with the SP fraction of samples is in agreement with that of the detergent resistant membrane portions [26], showing an enrichment of SLs and low content of glycerophospholipids, such as PE compared to the pattern found in PNS or SN (supplementary Fig. 1b). PE is a typical lipid component of the inner layer of plasma membranes and its low content in SP, with respect to the content in SN and PNS, confirms the good separation of the plasma membrane outer layer from the inner layer by cell protein biotinylation followed by streptavidin precipitation.

**Fig. 3:**
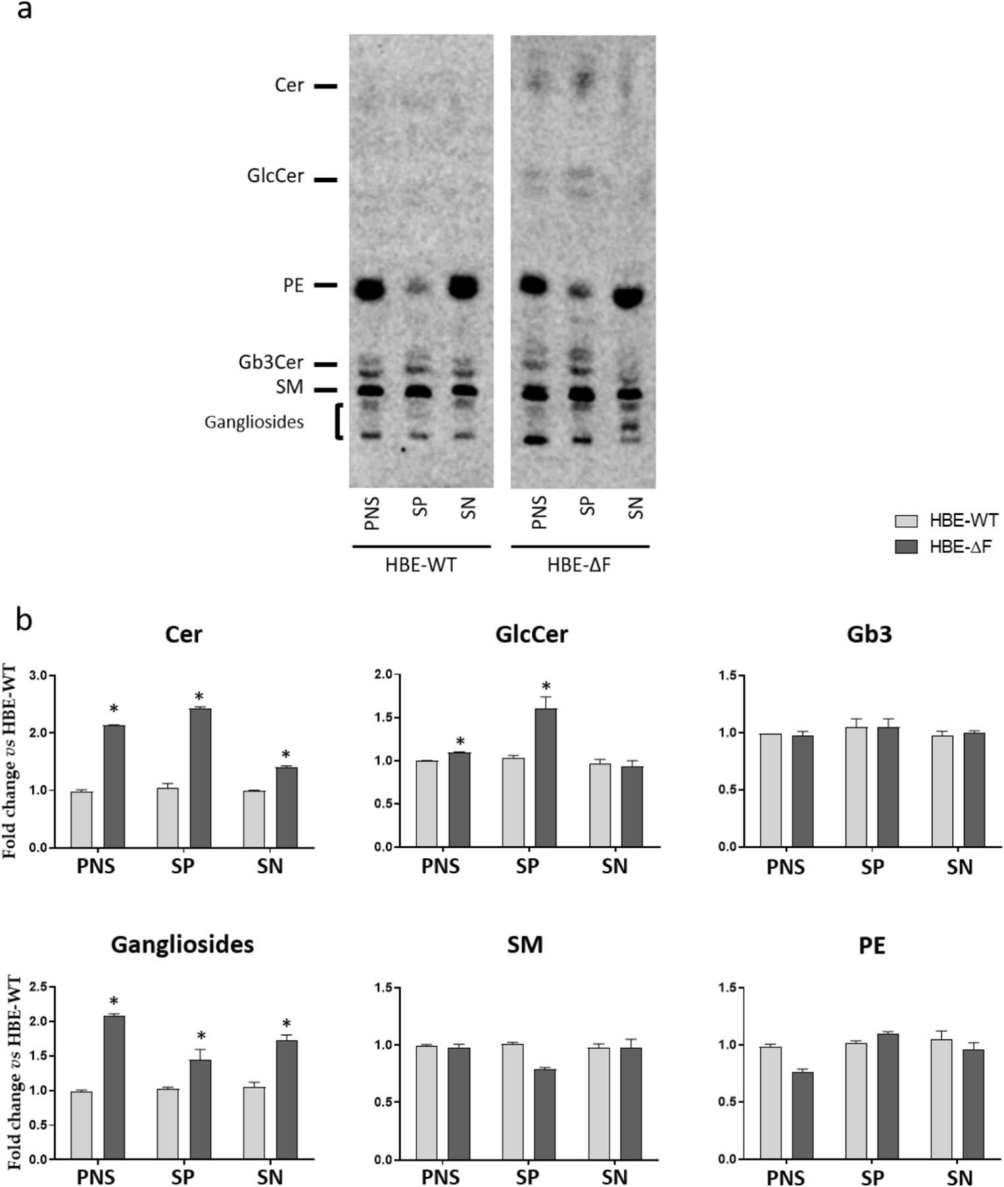
Lipid pattern of apical membrane enriched fractions from human primary bronchial epithelial cells differentiated at air-liquid interface. Cell sphingolipids were metabolically labeled at the steady state using [1-^3^H]sphingosine, PM proteins associated with the apical membrane were biotinylated and subjected to streptavidin-precipitation after cell lysis in lipid microenvironment preserving conditions. **a** Representative HPTLC of radioactive lipids in the post nuclear supernatant(PNS), streptavidin-precipitates (SP) and supernatant (SN) obtained after streptavidin-precipitation of human primary bronchial epithelial cells differentiated at air-liquid interface derived from healthy subjects (HBE-WT) and CF patients homozygous for F508del mutation (HBE-ΔF). **b** Semi quantitative graphs of sphingolipid species. Data are reported as fold change defined as the ratio between the percentage of radioactivity associated with each sphingolipid in HBE-ΔF cells and HBE-WT. Cer: ceramide, GlcCer: glucosylceramide, Gb3: globotriaosylceramide, SM: sphingomyelin, PE: phosphatidylethanolamine *p < 0.05 vs. HBE-WT

Of interest are the differences between the lipids associated with the apical membrane of HBE-WT and HBE-ΔF cells. As shown in Fig. 3, the apical membrane of HBE-ΔF is enriched in Cer and glucosylceramide with respect to that of HBE-WT. Of note, several lines of evidence suggest that the high hydrophobicity of these molecules and the ability to establish a strong hydrogen bond network could induce changes in the biophysical properties of biological membranes that can affect the activity of proteins altering specific signalling pathways. In addition, alterations in membrane biophysical properties might also influence the internalization, trafficking and sorting of lipids, proteins, drugs and even pathogens. Based on these considerations, the increased content of these lipids in fractions enriched in the apical membrane of HBE-ΔF cells leads to speculate that apical membrane of CF cells is characterized by a more rigid structure that could compromise its function [28, 29]. Moreover, the presence of high levels of Cer and glucosylceramide supports the hypothesis that the proinflammatory stimuli are triggered directly from the luminal side of the cell that is exposed to the possible infection and to cells of the immune system that are recruited to the infection site.

**Fig. 4:**
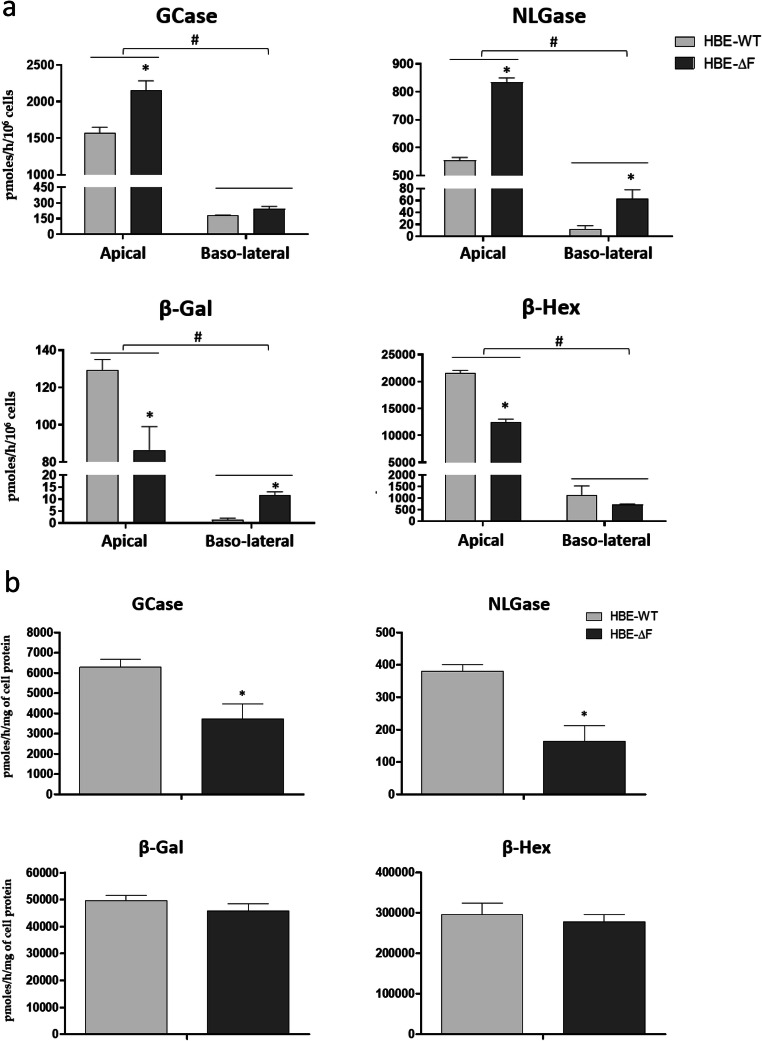
Glycohydrolases activity of human primary bronchial epithelial cells differentiated at air-liquid interface derived from healthy subjects and CF patients. **a **PM-associated activities of: β-glucocerebrosidase (GCase), non-lysosomal glucosylceramidase (NLGase), β-galactosidase (β-Gal), and β-hexosaminidase (β-Hex) were evaluated in the apical and basolateral membranes of human primary bronchial epithelial cells differentiated at air-liquid interface derived from healthy subjects (HBE-WT) and CF patients homozygous for F508del mutation (HBE-ΔF). Activities were expressed as pmoles/h/10^6^ cells. *p < 0.0001 vs. HBE-WT, ^#^p < 0.00001 vs. the indicated counterpart. **b** Total cell associated hydrolases activity of human primary bronchial epithelial cells differentiated at air-liquid interface derived from healthy subjects (HBE-WT) and CF patients homozygous for F508del mutation (HBE-ΔF). The measurements of the hydrolases activity were conducted on cell lysate and expressed as pmoles of product/mg of proteins per hour. *p < 0.002 vs. HBE-WT

To further verify this aspect, we studied the activity of the enzymes involved in the *in situ* production of Cer and glucosylceramide at the outer leaflet of the apical and basolateral membrane of HBE-WT and HBE-ΔF cells, differentiated under ALI. Indeed, it has been found in several cellular models that the fine tuning of SLs is directly triggered at the PM level by the action of several enzymes involved in their metabolism [30, 31]. To this purpose, we apply a methodology based on the use of methylumbelliferyl conjugated artificial substrates, that allows to detect specifically the activities of the hydrolases associated with the cell surface of living cells. In addition, to be sure to correctly distinguish between the activities associated with the external leaflet of the apical membrane with respect to the basolateral one, controls experiments were performed. Hence, we verified that substrates and the methylumbelliferone produced cannot cross the epithelium ensuring the hydrolysis of the substrates on both the sides of the polarized cells. As shown in Fig. 4, the activity of the PM-associated β-glucocerebrosidase (GCase), non-lysosomal β-glucosylceramidase (NLGase), β-galactosidase (beta-gal) and β-hexosaminidase are asymmetrically distributed, being higher on the external side of the apical membrane with respect to the basolateral one, with some differences between differentiated HBE-WT and HBE-ΔF cells (Fig. 4a). Interestingly, the activities of GCase and NLGase measured at the apical membranes in HBE-ΔF cells increased 1.4 and 1.5 fold respectively compared to the same activities in the apical membrane of HBE-WT. On the contrary, the activities of β-Hex and β-Gal at the apical membrane are decreased. Finally, the activities of NLGase and β-Gal at the basolateral membrane increased 8 and 10-fold in HBE-ΔF vs. HBE-WT.

Of note, when we measured the enzymatic activities in the total cell lysates, we did not find the same differences observed at the cell surface of HBE-WT and HBE-ΔF. In particular, β-Gal and β-Hex activities are similar, whereas GCase and NLGase are higher in HBE-WT cells. This indicates that the increase in the activities of GCase and NLGase observed in HBE-ΔF cells is restricted to the apical membrane (Fig. 4b).

These data suggest that a higher rate of SLs hydrolysis occurring at the PM may contribute to the Cer enrichment observed in CF cells. This consideration is in accordance with previous data demonstrating that the inhibition of the non-lysosomal β-glucosylceramidase NLGase has an antinflammatory effect in CF bronchial cells [14, 15].

## Methods

### Cells culture

Human primary bronchial epithelial cells (HBE) deriving from non-CF subjects (HBE-WT) and from CF patients homozygous for F508del mutation (HBE-∆F) are obtained from “Servizio Colture Primarie” of the Italian Cystic Fibrosis Research Foundation. These cells were grown as described in [19]. After a proliferative stage, cells were plated in transwell supports and induced to differentiation by the use of a differentiating medium. Cells at air-liquid interface were used after 14–16 days from the transwell seeding, once reached the complete differentiation.

### Sphingolipids analysis

Cell sphingolipids were analysed after their metabolic labelling at the steady state by the use of [1-^3^H]sphingosine as previously described [15]. Briefly, [1-^3^H]sphingosine was dissolved in the appropriate culture medium at a final concentration of 30 nM and administered to the cells for 2 h (pulse). In case of ALI culture, radioactive sphingosine was administered to both the apical and baso-lateral sides. After that, medium containing tritiated sphingosine was removed and cells were maintained in culture for further 48 h (chase). Cells were then collected in water and lyophilized. Total lipids from lyophilized cells were extracted with chloroform: methanol: water 20:10:1 by vol, followed by a second extraction with chloroform: methanol 2:1 by vol. The radioactivity associated with total lipid extract was evaluated by liquid scintillation, using a beta-counter system (Perkin-Elmer).

[^3^H]SLs were separated by high performance thin layer chromatography (HPTLC) using the solvent system chloroform: methanol: water 110:40:6 by vol, visualized by digital autoradiography using a BetaIMAGER™ TRacer system (Biospace Lab) and quantified with M3Vision software. The lipid identification was performed using purified radioactive standards [32].

### Enzymatic activity in total cell lysates

The enzymatic activities of β-glucocerebrosidase (GCase), non-lysosomal β-glucosylceramidase (NLGase), β-galactosidase (β-Gal), β-hexosaminidase (β-Hex) were determined in the total cell lysates using fluorogenic substrates, as previously described with few modifications [33, 34].

Briefly, cells were washed twice with PBS, harvested and suspended in water in the presence of a protease inhibitor cocktail (Sigma-Aldrich). Total cell proteins were quantified using DC™ Protein Assay (Biorad) according to the manufacturer’s instructions.

Equal amounts of cell proteins were transferred into a 96-well microplate and the assay was performed 3-fold in replicate. MUB-Glc was solubilized in McIlvaine buffer (pH 6) at concentration of 6 mM. MUB-Gal and MUG was solubilized in McIlvaine buffer (pH 5.2) at concentration of 500 µM.

In order to distinguish between GCase and NLGase activity, cell proteins were pre-incubated for 30 min at room temperature in McIlvaine buffer (pH 6) with 5 nM AMP-dNM [adamantane-pentyl-dNM; N-(5-adamantane-1-yl-methoxy-pentyl) deoxynojirimycin], a specific inhibitor of NLGase, and 1 mM CBE (Conduritol-B-epoxide) (Sigma), a specific inhibitor of GCase [35].

The reaction mixtures were incubated at 37 °C under gentle shaking. After different time points of incubation, 10 µl of the reaction mixtures were transferred to a 96 black well microplate adding 190 µl of glycine (0.25M, pH 10.7). Fluorescence was detected at different time points by microplate reader (Victor- Perkin Elmer).

Standards free MUB was used to construct calibration curve to quantify substrate hydrolysis. The enzymatic activity was expressed as pmoles of product/ hour/ mg proteins.

### Plasma membrane enzymatic activity

β-galactosidase (β-Gal), β-glucocerebrosidase (GCase), non-lysosomal β-glucosylceramidase (NLGase), β-hexosaminidase (β-Hex) activities associated with the external PM leaflet were assessed in living cells by a high throughput cell lived-based assay (HTA), as previously described with some modification [36, 37]. In the case of cells differentiated at ALI we used a snapwell support and plasma membrane enzymatic associated activities were measured in both the apical- and basolateral- membranes.

Culture medium was removed and cells were washed two times with DMEM-F12 without phenol red.

β-Gal, β-Hex activities were assayed using the artificial substrates 4-methylumbelliferyl-β-D-galactopyranoside (MUB-Gal), and 4-methylumbelliferyl-*N*-acetyl-β-D-glucosaminide (MUG) solubilized in DMEM-F12 without phenol red at pH 6, with final concentrations of 250 µM and 2 mM, respectively.

In order to distinguish between GCase and NLGase activity, cells were pre-incubated for 30 min at room temperature in DMEM-F12 containing 5 nM AMP-dNM [adamantane-pentyl-dNM; N-(5-adamantane-1-yl-methoxy-pentyl) deoxynojirimycin] a specific inhibitor of NLGase or 1 mM CBE (Condutiro-B-epoxide) (Sigma) a specific inhibitor of GCase [35]. We added medium with inhibitors both at the upper and bottom side of the chamber.

After that, medium containing the solubilized substrates and inhibitors were administer to the cells and incubated at 37 °C under gently stirring for 2 and 4 h.

At different time points, aliquots of medium (10 µl) were transferred in a 96 black well microplate and analyzed by a microplate reader (Victor, Perkin Elmer) (MUB: λex: 355 nm /λem: 460 nm), after adding 190 µl of 0.25 M glycine, with a pH of 10.7.

Standard free MUB was used to construct calibration curve to quantify substrate hydrolysis. The enzymatic activity was expressed as pmoles of product / hour/ 10^6^ cells.

### Isolation of apical membrane fraction

HBE cells fully differentiated at ALI were fed with 30 nM [1-^3^H]sphingosine [38] for 2 h (pulse) followed by 48 h chase to metabolically label all cellular SLs at the steady state [15]. At the end of the incubation, cells were washed with PBS and then incubated from the apical side with 1 mg/ml of EZ-Link™ Sulfo-NHS-Biotin (Thermo Fisher Scientific, Waltham, MA, USA) in PBS, pH 7.4 (5 ml/ T75 flasks) for 30 min at 4 °C [39]. Under these experimental conditions the internalization of the biotin derivative does not occur and biotinylation is restricted to the cell apical surface proteins [40]. After biotin labeling, cells were rinsed twice with 100 mM glycine to remove the excess of unbounded biotin and then washed with ice-cold PBS. Cells were mechanically harvested in PBS and centrifuged at 270 *g* for 10 min. Cell pellet was lysed in 1 ml of 1% Triton X-100 in TNEV buffer (10 mM Tris-HCl, 150 mM NaCl, 5 mM EDTA, pH 7.5) supplemented with Protease Inhibitor Cocktail (Sigma-Aldrich, St. Louis, MO, USA) and 1 mM Na_3_VO_4_ (Sigma-Aldrich) for 20 min in ice, and then homogenized with Dounce homogenizer (tight pestle). Cell lysates were centrifuged at 4 °C for 5 min at 800 *g* to remove nuclei and cellular debris. The resulting post nuclear supernatants (PNS) of HBE-WT and HBE-∆F (corresponding to 1 mg protein of total cell lysate) were precipitated using 200 µL Dynabeads™ M-280 Streptavidin (Thermo Fisher Scientific). The mixtures were stirred overnight at 4 °C and then centrifuged to recover the biotinylated proteins in the pellet (SP) by magnet. Under these experimental conditions (domain-preserving conditions), we preserved the organization of lipid domains [41, 42]. Radioactive lipids from the pellet fractions were extracted twice with 200 µL of chloroform/methanol 2:1 (v:v), while radioactive lipids from PNS and supernatant after streptavidin-precipitation (SN) were extracted following the procedures described above for the sphingolipids and radioactive analyses.

### Western blotting

Proteins of PNS, SN, SP samples were analysed by SDS-PAGE under reducing conditions. Equal ratio in terms of volume of samples PNS, SN and SP were separated by 4–20% precasted polyacrylamide gradient gels and then transferred to PVDF membranes by electroblotting. Blotted biotinylated proteins were detected by HRP-Streptavidin incubation for 1 h at RT. β-actin was detected by immunoblotting with the primary antibody (BD) at 4 °C overnight, followed by incubation with HRP-secondary antibody and detection with a chemiluminescent kit (WESTAR ηC, Cyanagen, Bologna, Italy). Digital images were obtained by the chemiluminescence system Alliance Mini HD9 (UVItec, Cambridge, UK).

### Statistical analysis

All the experiments were performed in triplicate as differently indicated and statistical significance was determined by Student-Neumann-Keuls post-hoc test (comparison between two groups), and by one-way or two-way ANOVA (followed by Turkey or Dunnett Newman-Keuls on Bonferroni post test) for more than two groups, with statistical significance set at p < 0.05, using GraphPad Prism 8.

## Electronic supplementary material

ESM 1 (PDF 315 KB)

## Data Availability

We declare that all the row data are available.

## References

[1] Uemura K, Macher BA, DeGregorio M, Scudder P, Buehler J, Knapp W, Feizi T (1985). Glycosphingolipid carriers of carbohydrate antigens of human myeloid cells recognized by monoclonal antibodies. Biochim Biophys Acta (.

[2] Tivodar S, Paladino S, Pillich R, Prinetti A, Chigorno V, van Meer G, Sonnino S, Zurzolo C (2006). Analysis of detergent-resistant membranes associated with apical and basolateral GPI-anchored proteins in polarized epithelial cells. FEBS Lett..

[3] van IJzendoorn SCD, Agnetti J, Gassama-Diagne A (2020). Mechanisms behind the polarized distribution of lipids in epithelial cells. Biochim Biophys Acta Biomembr.

[4] Röper K, Corbeil D, Huttner WB (2000). Retention of prominin in microvilli reveals distinct cholesterol-based lipid micro-domains in the apical plasma membrane. Nat. Cell Biol..

[5] Hansen GH, Pedersen J, Niels-Christiansen LL, Immerdal L, Danielsen EM (2003). Deep-apical tubules: dynamic lipid-raft microdomains in the brush-border region of enterocytes. Biochem. J..

[6] Yu RK, Nakatani Y, Yanagisawa M (2009). The role of glycosphingolipid metabolism in the developing brain. J Lipid Res.

[7] Hanqing M, Avrova N, Månsson JE, Molin K, Svennerholm L (1986). Gangliosides and neutral glycosphingolipids of normal tissue and oat cell carcinoma of human lung. Biochim. Biophys. Acta.

[8] Lee JW, Ryu JY, Yoon G, Jeon HK, Cho YJ, Choi JJ, Song SY, Do IG, Lee YY, Kim TJ, Choi CH, Kim BG, Bae DS (2015). Sphingosine kinase 1 as a potential therapeutic target in epithelial ovarian cancer. Int J Cancer.

[9] Petrache I, Natarajan V, Zhen L, Medler TR, Richter AT, Cho C, Hubbard WC, Berdyshev EV, Tuder RM (2005). Ceramide upregulation causes pulmonary cell apoptosis and emphysema-like disease in mice. Nat Med.

[10] Medler TR, Petrusca DN, Lee PJ, Hubbard WC, Berdyshev EV, Skirball J, Kamocki K, Schuchman E, Tuder RM (2008). Petrache, I. Apoptotic sphingolipid signaling by ceramides in lung endothelial cells. Am J Respir Cell Mol Biol.

[11] Riordan, J.R., Rommens, J.M., Kerem, B., Alon, N., Rozmahel, R., Grzelczak, Z., Zielenski, J., Lok, S., Plavsic, N., Chou, J.L., et al.: Identification of the cystic fibrosis gene: cloning and characterization of complementary DNA. Science **245**(4922), 1066–73 (1989) 10.1126/science.2475911 2475911

[12] Bobadilla JL, Macek M, Fine JP, Farrell PM (2002). Cystic fibrosis: a worldwide analysis of CFTR mutations–correlation with incidence data and application to screening. Hum. Mutat..

[13] Dechecchi MC, Nicolis E, Mazzi P, Cioffi F, Bezzerri V, Lampronti I, Huang S, Wiszniewski L, Gambari R, Scupoli MT, Berton G, Cabrini G (2011). Modulators of sphingolipid metabolism reduce lung inflammation. Am J Respir Cell Mol Biol.

[14] Loberto N, Tebon M, Lampronti I, Marchetti N, Aureli M, Bassi R, Giri MG, Bezzerri V, Lovato V, Cantù C, Munari S, Cheng SH, Cavazzini A, Gambari R, Sonnino S, Cabrini G, Dechecchi MC (2014). GBA2-encoded β-glucosidase activity is involved in the inflammatory response to Pseudomonas aeruginosa. PLoS One.

[15] Schiumarini D, Loberto N, Mancini G, Bassi R, Giussani P, Chiricozzi E, Samarani M, Munari S, Tamanini A, Cabrini G, Lippi G, Dechecchi MC, Sonnino S (2017). Aureli, M. Evidence for the Involvement of Lipid Rafts and Plasma Membrane Sphingolipid Hydrolases in Pseudomonas aeruginosa Infection of Cystic Fibrosis Bronchial Epithelial Cells. Mediators Inflamm..

[16] Becker KA, Riethmüller J, Seitz AP, Gardner A, Boudreau R, Kamler M, Kleuser B, Schuchman E, Caldwell CC, Edwards MJ, Grassmé H, Brodlie M, Gulbins E (2018). Sphingolipids as targets for inhalation treatment of cystic fibrosis. Adv Drug Deliv Rev.

[17] Aureli M, Schiumarini D, Loberto N, Bassi R, Tamanini A, Mancini G, Tironi M, Munari S, Cabrini G, Dechecchi MC, Sonnino S (2016). Unravelling the role of sphingolipids in cystic fibrosis lung disease. Chem Phys Lipids.

[18] Mingione A, Dei Cas M, Bonezzi F, Caretti A, Piccoli M, Anastasia L, Ghidoni R, Paroni R, Signorelli P (2020). Inhibition of Sphingolipid Synthesis as a Phenotype-Modifying Therapy in Cystic Fibrosis. Cell Physiol Biochem.

[19] Scudieri P, Caci E, Bruno S, Ferrera L, Schiavon M, Sondo E, Tomati V, Gianotti A, Zegarra-Moran O, Pedemonte N, Rea F, Ravazzolo R, Galietta LJ (2012). Association of TMEM16A chloride channel overexpression with airway goblet cell metaplasia. J. Physiol..

[20] Garcia-Castillo MD, Chinnapen DJ, Lencer WI (2017). Membrane Transport across Polarized Epithelia. Cold Spring Harb. Perspect. Biol..

[21] Grassmé H, Carpinteiro A, Edwards MJ, Gulbins E, Becker KA (2014). Regulation of the inflammasome by ceramide in cystic fibrosis lungs. Cell Physiol Biochem.

[22] Kovacic B, Sehl C, Wilker B, Kamler M, Gulbins E, Becker KA (2017). Glucosylceramide Critically Contributes to the Host Defense of Cystic Fibrosis Lungs. Cell Physiol Biochem.

[23] Hajj R, Baranek T, Le Naour R, Lesimple P, Puchelle E, Coraux C (2007). Basal cells of the human adult airway surface epithelium retain transit-amplifying cell properties. Stem Cells.

[24] Nitta, T., Kanoh, H., Inamori, K.I., Suzuki, A., Takahashi, T., Inokuchi, J.I. Globo-series glycosphingolipids enhance Toll-like receptor 4-mediated inflammation and play a pathophysiological role in diabetic nephropathy. Glycobiology: (2019) 10.1093/glycob/cwy105 10.1093/glycob/cwy105 30476082

[25] Sonnino S, Prinetti A, Mauri L, Chigorno V, Tettamanti G (2006). Dynamic and structural properties of sphingolipids as driving forces for the formation of membrane domains. Chem. Rev..

[26] Aureli M, Grassi S, Sonnino S, Prinetti A (2016). Isolation and Analysis of Detergent-Resistant Membrane Fractions. Methods Mol. Biol..

[27] Delorme-Axford E, Coyne CB (2011). The actin cytoskeleton as a barrier to virus infection of polarized epithelial cells. Viruses.

[28] Ventura AE, Mestre B, Silva LC (2019). Ceramide Domains in Health and Disease: A Biophysical Perspective. Adv. Exp. Med. Biol..

[29] Varela AR, Ventura AE, Carreira AC, Fedorov A, Futerman AH, Prieto M, Silva LC (2016). Pathological levels of glucosylceramide change the biophysical properties of artificial and cell membranes. Phys. Chem. Chem. Phys..

[30] Aureli M, Loberto N, Bassi R, Ferraretto A, Perego S, Lanteri P, Chigorno V, Sonnino S, Prinetti A (2012). Plasma membrane-associated glycohydrolases activation by extracellular acidification due to proton exchangers. Neurochem Res.

[31] Aureli M, Gritti A, Bassi R, Loberto N, Ricca A, Chigorno V, Prinetti A, Sonnino S (2012). Plasma membrane-associated glycohydrolases along differentiation of murine neural stem cells. Neurochem Res.

[32] Loberto N, Prioni S, Bettiga A, Chigorno V, Prinetti A, Sonnino S (2005). The membrane environment of endogenous cellular prion protein in primary rat cerebellar neurons. J. Neurochem..

[33] Aureli M, Masilamani AP, Illuzzi G, Loberto N, Scandroglio F, Prinetti A, Chigorno V, Sonnino S (2009). Activity of plasma membrane beta-galactosidase and beta-glucosidase. FEBS Lett..

[34] Overkleeft HS, Renkema GH, Neele J, Vianello P, Hung IO, Strijland A, van der Burg AM, Koomen GJ, Pandit UK, Aerts JM (1998). Generation of specific deoxynojirimycin-type inhibitors of the non-lysosomal glucosylceramidase. J. Biol. Chem..

[35] Ridley CM, Thur KE, Shanahan J, Thillaiappan NB, Shen A, Uhl K, Walden CM, Rahim AA, Waddington SN, Platt FM, van der Spoel AC (2013). β-Glucosidase 2 (GBA2) activity and imino sugar pharmacology. J. Biol. Chem..

[36] Aureli M, Prioni S, Mauri L, Loberto N, Casellato R, Ciampa MG, Chigorno V, Prinetti A, Sonnino S (2010). Photoactivable sphingosine as a tool to study membrane microenvironments in cultured cells. . J Lipid Res.

[37] Scandroglio F, Venkata JK, Loberto N, Prioni S, Schuchman EH, Chigorno V, Prinetti A, Sonnino S (2008). Lipid content of brain, brain membrane lipid domains, and neurons from acid sphingomyelinase deficient mice. J. Neurochem..

[38] Toyokuni T, Nisar M, Dean B, Hakomori S-I (1991). A facile and regiospecific tritiation of sphingosine: synthesis of (2S,3R,4E)-2-amino-4-octadecene-1,3-diol-1-3H. J. Labelled Comp. Radiopharm.

[39] Altin JG, Pagler EB (1995). A one-step procedure for biotinylation and chemical cross-linking of lymphocyte surface and intracellular membrane-associated molecules. Anal. Biochem..

[40] Cole SR, Ashman LK, Ey PL (1987). Biotinylation: an alternative to radioiodination for the identification of cell surface antigens in immunoprecipitates. Mol Immunol.

[41] Prinetti A, Prioni S, Chigorno V, Karagogeos D, Tettamanti G, Sonnino S (2001). Immunoseparation of sphingolipid-enriched membrane domains enriched in Src family protein tyrosine kinases and in the neuronal adhesion molecule TAG-1 by anti-GD3 ganglioside monoclonal antibody. J. Neurochem..

[42] Brown DA, Rose JK (1992). Sorting of GPI-anchored proteins to glycolipid-enriched membrane subdomains during transport to the apical cell surface. Cell.

[43] Aureli M, Loberto N, Lanteri P, Chigorno V, Prinetti A, Sonnino S (2011). Cell surface sphingolipid glycohydrolases in neuronal differentiation and aging in culture. . J Neurochem.

