# Supplementary material for: Sphingolipids and plasma membrane hydrolases in human primary bronchial cells during differentiation and their altered patterns in cystic fibrosis
Source: Glycoconj J. 2020 Jul 14;37(5):623–33. doi: 10.1007/s10719-020-09935-x (PMC7501107; doi:10.1007/s10719-020-09935-x)
Supplement: Supplementary file 1 — (PDF 315 KB) [file 10719_2020_9935_MOESM1_ESM.pdf]

Figure

Supplementary Figure 1

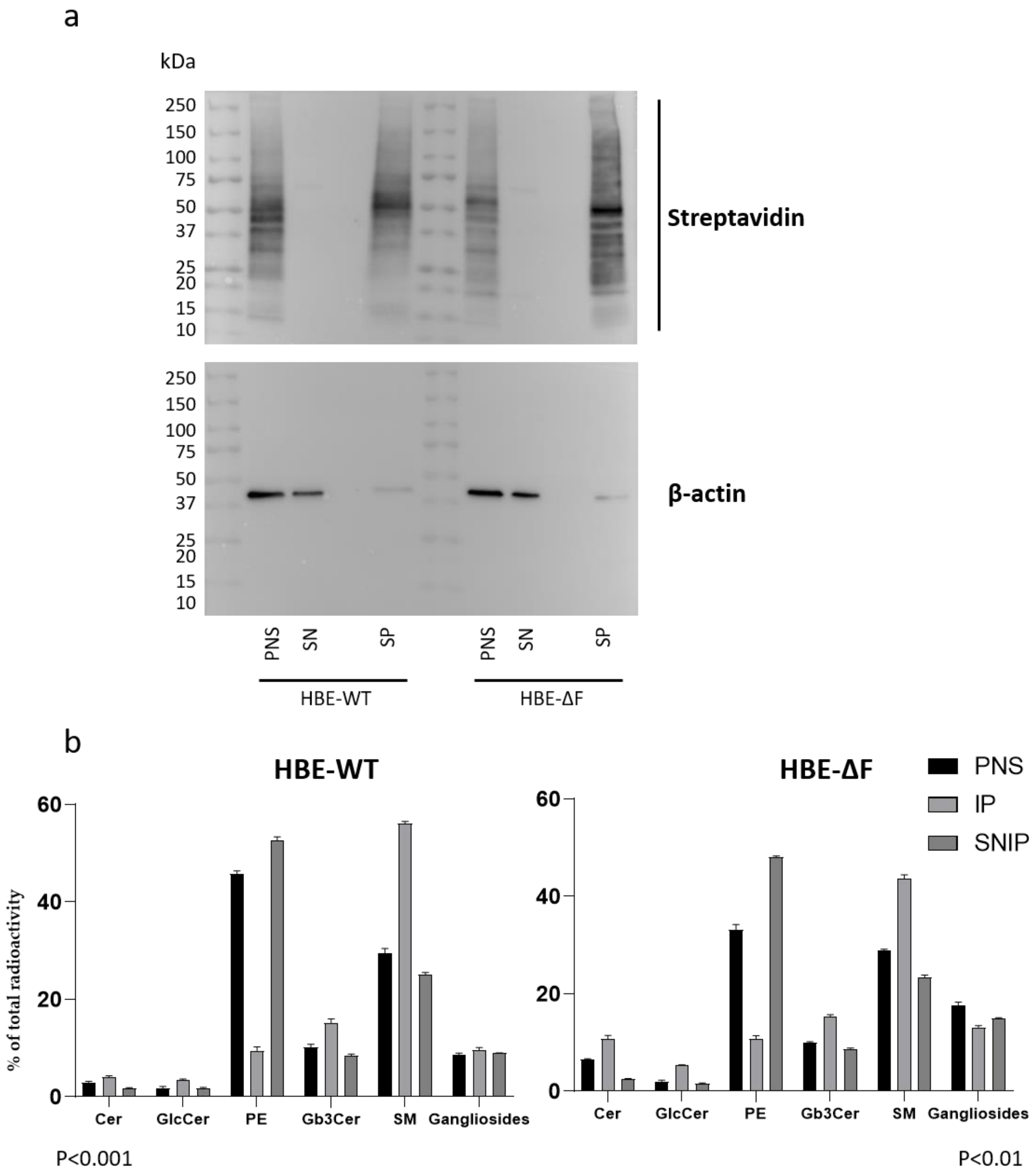

Supplemental Figure 1. Evaluation of the streptavidin precipitation efficiency.

a) Representative staining of biotinylated apical membrane proteins (upper panel) and of β-actin (lower panel) in post-nuclear supernatants (PNS), supernatants (SN) and streptavidin-precipitates (SP) obtained from human primary bronchial epithelial cells differentiated at air-liquid interface derived from healthy subjects (HBE-WT) and CF patients homozygous for F508del mutation (HBE-ΔF). **b**) Semi quantitative graph of radioactive sphingolipid species described in figure 3, expressed as percentage with respect to total sphingolipids incorporated radioactivity in HBE-WT and HBE- ΔF, respectively
